# Shrapnel Ball Encysted on the Anterior Wall of the Ascending Colon

**Published:** 1918-05

**Authors:** 


**Shrapnel Ball Encysted on the Anterior Wall of the Ascending Colon.** Mauclaire, quoted in Arch. Med. d'Electricite, Sept., 1917, reports a case, operated on under electric light, switched off to use X-rays to locate the ball on the screen, and on again when the ball was firmly grasped after the preliminary incision and location. It was surrounded with false membrane and was excised without opening into the peritoneal cavity. (Note: Having had the results of intestinal adhesions, especially about the appendix stump and the caecum brought to our attention many times in civil practice and in a surprising number of cases in a brief military experience, where the genuineness of the complaints made by the patient is a serious problem not encountered in civil practice except in marked neurotic cases, the question may be raised whether it was wise to avoid the slight danger of peritoneal invasion by leaving the cicatricial tissue. Ed.)

